# Frame Reflection Lab: a Playful Method for Frame Reflection on Synthetic Biology

**DOI:** 10.1007/s11569-018-0318-9

**Published:** 2018-06-11

**Authors:** Marjoleine G. van der Meij, Anouk A. L. M. Heltzel, Jacqueline E. W. Broerse, Frank Kupper

**Affiliations:** 1 Athena Institute, VU University of Amsterdam, De Boelelaan 1085, Building 1081, Room S554, 1081 HV Amsterdam, The Netherlands; 2 Athena Institute, VU University of Amsterdam, De Boelelaan 1085, Building 1081, 1081 HV Amsterdam, The Netherlands; 3 Athena Institute, VU University of Amsterdam, De Boelelaan 1085, Building 1081, Room T511, 1081 HV Amsterdam, The Netherlands; 4 Athena Institute, VU University of Amsterdam, De Boelelaan 1085, Building 1081, Room S562, 1081 HV Amsterdam, The Netherlands

**Keywords:** Frame reflection, Reflection methods, Playful tools, Video-narratives, Responsible research and innovation, Synthetic biology

## Abstract

Synthetic biology is an emerging technology that asks for inclusive reflection on how people frame the field. To unravel how we can facilitate such reflection, this study evaluates the Frame Reflection Lab (FRL). Building upon playfulness design principles, the FRL comprises a workshop with video-narratives and co-creative group exercises. We studied how the FRL facilitated frame reflection by organizing workshops with various student groups. Analysis of 12 group conversations and 158 mini-exit surveys yielded patterns in first-order reflection (problem analysis and solution finding in reflection on the development of synthetic biology as a field) as well as patterns in second-order reflection (reflection on values and assumptions underlying the first-order reflection). Also patterns in participants’ (re)framing of synthetic biology could be induced; participants’ viewpoints converged to some extent, yet with openness to individual viewpoint differences. Although the FRL method fortified the reflection processes of participants, the narratives and the workshop’s flexible format could inhibit the reflection too. Therefore, we advise designers of future frame reflection methods to apply stronger conversational facilitation and narratives of slightly mysterious yet identifiable narrators, in case e.g. video-narratives are created and used to scaffold the reflection process. Nevertheless, we argue that the use of a playful frame reflection method like the FRL could function well as (1) a step to precede more application-specific deliberation or decision-making on synthetic biology and as (2) a method for the collection of contemporary citizen viewpoints plus rationales underlying these, for the further (societally) responsible development of the emerging field.

## Introduction

Emerging technologies such as artificial intelligence, nanotechnology, and biotechnology are shrouded in uncertainty: they will have positive effects on our society, but will also have undesirable, unintended, and unpredictable impacts [1]. There are technical uncertainties, for instance, regarding safety and security, but also uncertainties concerning social and ethical impacts (e.g., in [2, 3]). As a result, there are many ways to problematize emerging technologies and to envisage how they should develop [2]. This range of eligible viewpoints about emerging technologies can lead to heated discussions [2]. To deal with such potential controversy, there is a need for processes that enable the socially responsible development of emerging technologies [1, 4].

To assist such development of emerging technologies, a new policy narrative has emerged in the last few years, known as “Responsible Research and Innovation” (RRI) [1, 5, 6]. With inclusion, anticipation of impacts, reflexivity, and responsiveness as core process dimensions [4], RRI aims to achieve research and innovation outcomes that are socially desirable, sustainable, and ethically acceptable [6]. To realize the RRI process dimensions, RRI scholars emphasize that it is important to facilitate reflection on technical and ethical aspects, purposes, motivations, “intended and unintended impacts, consequences and implications” ([1], p. 754), but also on “tacit understandings, assumptions” and “framings” ([4], p. 1575)*.* This reflection should “inclusively open up […] to broad, collaborative deliberation through processes of dialogue, engagement and debate, inviting and listening to wider perspectives from publics and diverse stakeholders” ([1], p. 755). In other words, a crucial element of RRI is the continuous engagement of various actors in a reflexive dialogue on diverse viewpoints regarding research and innovation. In this study, we refer to these dialogues as *RRI reflection*.

It is no easy matter to organize RRI reflection on emerging technologies. First, by definition, emerging technologies often have only a few concrete applications that have been realized, or applications are described only as potentially promising concepts [7]. Their status as emerging makes it hard for most people to understand emerging technologies and anticipate their potential impacts (ibid.). Second, it is a challenge to create a safe environment in which participants feel able to voice their diverse and potentially conflicting viewpoints [8, 9]. Third, while a growing body of literature focuses on the conceptualization of RRI [10], there is little literature on its practical realization on which to build RRI reflection. This calls for research into reflection methods for RRI that support people in making sense of emerging technologies and in paying attention to diverse sense-makings.

Van der Meij, Broerse, and Kupper [11] argued that playful tools have great potential to support RRI reflection. Playful tools can trigger participants’ *playfulness*, which is an attitude that makes people open to exploring and able to cope with complex tasks like reflection on emerging, potentially controversial, technologies (ibid.). Activating such playfulness during RRI reflection processes requires the embedding of one or more playfulness activity principles in the design of playful reflection methods, as well as the application of playfulness processes conditions (ibid.). Examples of such activity principles are *narration*, *imagination*, and *co-creation* (ibid.). *Narration* refers to the use of (personal) stories for reflection. Presenting stories of personal experiences and perspectives enables story-receivers to put themselves in the shoes of storytellers, which encourages them to pay in-depth attention to viewpoints that are similar to or differ from their own [12]. Another activity principle that is useful for RRI reflection is *imagination*, which comprises posing questions or providing visual–spatial objects during the reflection process that allow for ambiguous interpretation [11]. This ambiguity fosters developing new perspectives. *Co-creation* refers to RRI reflection activities by which participants collaboratively create objects, visualizations or scenarios (ibid.). The negotiation during such activities can trigger understanding of the existence of different viewpoints, agreement to disagree, and alignment in thinking (ibid.). Examples of playfulness-simulating process conditions are (1) *stimulating guidance*, such as encouraging and rewarding participation; (2) a clear *focus*, covering one particular sub-topic at a time and providing a thought-out step-wise outline for the reflection process; and (3) offering *experimentation space* for testing multiple ideas and actions, in which revising previous ways of thinking and doing is encouraged and not seen as a mistake [11].

Several scholars have described playful tools, formats, methods, and environments for reflection on various research and innovation fields (e.g., in [7, 13–17]), but have paid minimal attention to explicitly linking the designs of the tools, formats, methods, or environments to the intended reflection [7]. To investigate how playful methods can be designed for RRI reflection, this study evaluates a playful method called *Frame Reflection Lab* (FRL hereafter). The FRL aims to support researchers and non-researchers in reflection on synthetic biology. Synthetic biology refers to the use of biotechnology in creating new biological products and systems, with specific preconceived functionalities (cf. [18]). Currently targeted application domains are, for instance, health care, food production, and renewable energy. While only a few applications of synthetic biology have been realized to date (see, e.g., in [19, 20]), the field is (potentially) controversial: there are debates on the field’s safety and security, as well as regarding the field’s potential impacts on the environment and social structures, such as (in)equality [21–23]. Synthetic biology therefore serves as an interesting emerging technology for the purpose of this study.

The FRL method consists of a 90-min workshop, in which participants reflect on framings of what synthetic biology is (step 1), deeper beliefs underlying various views (step 2), and approaches to guide the further development of the field (step 3). Built on playfulness design elements of Van der Meij et al. [11], as described above, the FRL applies playfulness activity principles as follows:

- Video-*narratives* of four semi-fictitious narrators, with each a different framing of synthetic biology, for each workshop step
- *Co-creation* exercises on an A2 canvas, in groups of up to seven people, to collaboratively analyze the narratives and elicit their own perspectives
- *Imagination*-triggering cards to support the reflection

The FRL includes a number of playfulness process conditions as well (cf. [11]). First, there is a plenary workshop facilitator, a social scientist, who is not personally active in the synthetic biology field. This facilitator instructs and encourages participants throughout each workshop step and thereby provides *stimulating guidance* to participants in their reflection. Furthermore, several other facilitators freely walk around, also social scientists, to ensure that participants *focus* on the given conversation topic (different in each workshop step), and guide the *experimentation space* by encouraging people to test different ways of thinking.

We tested the FRL in various settings with (homogeneous) student groups, namely international Bachelor, Master, and PhD students engaged in iGem, a competition about synthetic biology; Dutch high-school students aged 16–18, taking a biology specialization; and Master students at a university in the Netherlands specializing in Management, Policy & Entrepreneurship, and Global Health (hereafter *Master students*). By evaluating these test workshops, we aimed to answer the following research question: *In what ways does the FRL trigger frame reflection?*

## Frame Reflection and Playfulness

RRI reflection requires the exchange of views regarding research and innovation between people from different backgrounds. The diversity of people included in such processes poses a challenge. Initially, people may have completely different viewpoints regarding complex issues or situations [24], for instance on safety, such as *Who is allowed to use biotechnological tools to create new biological systems?* or on (in)equality, such as *Will synthetic biology-based health care be accessible for only the rich?* This can result in seemingly intractable situations, like an impasse in conversations about *how synthetic biology should develop as a field*, and *what policies should guide that development.* Such an impasse often stems from people’s different ways of framing a new phenomenon (ibid.). In this section, therefore, we conceptualize frames, framing, and frame reflection for the purpose of this study.

In general, a person’s view of a new and complex issue arises from a process of sense making [25], which is an interactive and social process. In that process, we select aspects of the new phenomenon that we can relate to what we already know [24, 26]. This enables us to form a tentative view of the issue, which is called a *frame* [24–26]. Through interaction with others, we re-define and further shape our frame [27]. This interactive process, through which the frame is negotiated and (re)shaped, is referred to as *framing* [25]. In terms of synthetic biology framing, for example, Ancillotti et al. [23] found that some people see synthetic biology as a potentially dangerous field and argue that caution, rules, and regulations should be applied to assure the socially and ethically responsible development of the field. Other people, however, consider synthetic biology to be an incredibly interesting field full of opportunities to tackle contemporary world problems and argue that total freedom should be given to people for the discovery of truly innovative applications (ibid.).

When people have to reflect and negotiate about how to tackle a particular issue, *frame reflection* is useful [25]. Frame reflection means that people engaged in a negotiation reflect on how their framing leads to the rise of differences in opinion regarding the most appropriate solution to a given issue (ibid.). It requires revealing arguments that underlie these differences (ibid.). Grin and Van der Graaf described this process as a policy learning process and made a useful distinction between first- and second-order reflection [28]. People often initially employ first-order reflection, being the “evaluation of the effectiveness, including unintended side-effects, and costs of alternative solution strategies for the achievement of the objectives set,” and “defining the problem in the case at hand,” after which “policy objectives and the consequent causal chains of means and ends chosen to implement them, are validated as contributions to solving that problem” ([28], p. 299). To prevent conversations between people with very different viewpoints from leading to an impasse, argumentation underlying the opinions, called second-order discourse, is ideally also revealed (ibid.). This second-order reflection comprises the exchange of “fundamental preferences about the social order” and evaluation of “systems of values and perceptions” ([28], p. 300). Schön and Rein [25] call for a comparable reflection process to deal with differences, specifically the exchange of assumptions and values, in order to lead to mutual understanding among those participating in a negotiation.

Extrapolating this first- and second-order problem analysis and solution-finding process to reflection on an emerging field like synthetic biology, frame reflection then comprises:

- Eliciting viewpoints on the effectiveness of the field, its side effects, and comparison with alternative technologies in the light of their purposes
- Discovery of values and assumptions that underlie these viewpoints
- Defining solution strategies to deal with identified issues regarding the field’s effectiveness and potential effects, like laws or (joint) decision-making structures to guide its further development

In scholarly literature, playful methods have made promising contributions to such reflection on emerging technologies, albeit not explicitly described as such (e.g., in [7, 13–17, 29]). For example, Cox et al. [13] developed and evaluated a theatrical play for reflection on genetics, which could be seen as a practical example of playfulness design principle *narration* [11]. An alternative form of narration could be found in van der Meij et al. [30], who developed and evaluated video-narratives for reflection on synthetic biology. *Narration* in combination with an element of *imagination* [11] can be found in Schmidt et al. [29]. These authors describe the film competition Bio-fiction, which entails a festival and screenings around the globe to facilitate reflection on synthetic biology (ibid.).

Horst and Michael [15] studied other playfulness design elements, in addition to *narration* and *imagination*. Although not explicitly referred to as such, the design of the biotechnology exhibition that they evaluate gave visitors the opportunity to contribute to the content by writing notes in response to a poll question *who should control research in biotechnology?*, so that visitors would be stimulated to discuss the various answers to this question with their co-visitors [15]. This could be considered as an interesting example of playfulness activity principle *co-creation* [11]. In addition, visitors were allowed to experiment freely with the objects of another exhibit, allowing them to discover and discuss different ways of doing and seeing in a more open and intuitive manner [15]. In this way, Horst and Michael’s exhibit design also seems to apply playfulness process condition *experimentation space*, which calls for a non-judgmental environment that allows the discovery of diverse ways of doing and looking at a particular issue [11].

Going further than *narration*, *imagination*, *co-creation*, and *experimentation space*, Felt et al. [7] described an inspiring method for reflection on nanotechnology, using cards about its potential applications, issues and futures. They concluded that the cards and debate enabled citizens to reflect purposefully on the field, yet the debate could have been deeper and the cards alone did not guarantee equal opportunity to participate. Their reflection illustrates that providing what Van der Meij et al. call *focus* (e.g., with the help of cards and specific tasks) but also *stimulating guidance*, e.g., by facilitation that promotes equal participation [11], are useful process design conditions for a reflection tool to fulfill the potential of playful activity principles for reflection.

Felt et al. [7], Cox et al. [13], Horst and Michael [15], and Schmidt et al. [29] all illustrate that their methods, embedding various tools and formats comparable to playfulness design elements (cf. [11]), make promising contributions to reflection on research and innovation. These studies do not, however, cover frame reflection explicitly. As van der Meij et al. [30] noted that second-order reflection regarding research and innovation in particular needs more than a (video-)narrative and a conversation, we argue that a playful RRI reflection method for frame reflection on synthetic biology may require more extensive application of playfulness process conditions *stimulating guidance*, *focus*, and *experimentation space*, and also activity principles *co-creation* and *imagination* [cf. 11]. In the next section, we elaborate on the FRL design, which aimed to put this into practice.

## Research Methodology

### The FRL Design

**Fig. 1:**
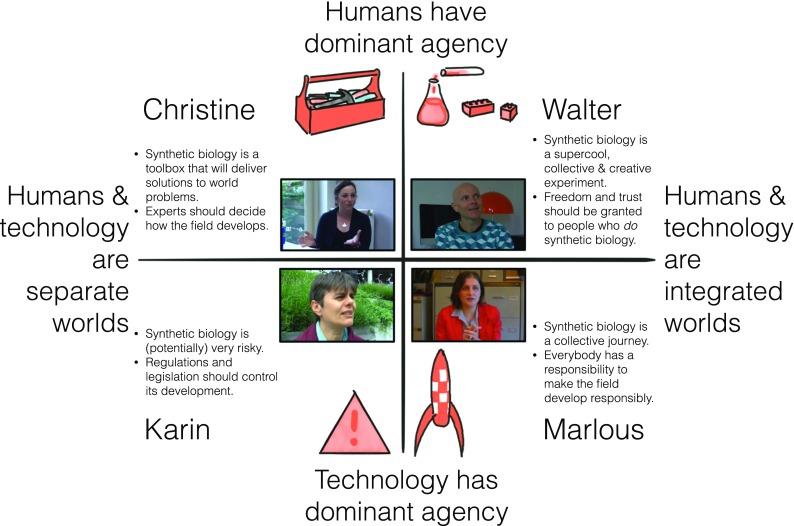
The narrators Christine, Karin, Walter, and Marlous and their synthetic biology framing characteristics

As briefly indicated above, the FRL comprises a method for facilitated workshops with video-narratives and several co-creation group exercises. The video-narratives are based on research into citizens’ views on synthetic biology [31], research into the relationship between technology and society [32], and a biotech viewpoint categorization described in Boerwinkel et al. [2]. They comprise narratives of four narrators named *Christine* (physicist), *Karin* (teacher), *Walter* (music producer), and *Marlous* (journalist) (see Fig. 1). Christine and Karin both externalize technology (cf. [32]): they see technology and human beings as separate worlds. Christine argues that human beings have dominant agency over technology (existentialist), whereas Karin argues that technology has dominant agency (determinist). Walter and Marlous represent a transhumanist view (cf. ibid.): they see human beings and technology as integrated worlds. Walter adheres to an existentialist version of this transhumanist view, whereas Marlous is a determinist-transhumanist. Christine sees synthetic biology as a toolbox that will provide solutions to world problems. Responsible development can be realized by letting experts undertake a risk assessment, for instance (cf. [2]). Karin considers synthetic biology to be (potentially) risky, because of its autonomous development (cf. ibid.), and calls for regulations and legislation to control its development. Conversely, Walter sees the field as a collective creative experiment, and grants freedom and trust to all people who undertake synthetic biology. Similarly, Marlous considers synthetic biology as a collective journey, but pays more attention to everyone’s responsibility to make the field develop responsibly, e.g., by engaging in dialogue and deliberation to co-decide on its future (cf. ibid.).

The narrators share their viewpoints in three separate clips:

1. *What is synthetic biology?* and *What is the impact of synthetic biology on society?*, representing the problematizing of the field in line with Grin and van der Graaf’s first-order reflection on effectiveness, goals, and impacts [28].
2. *What is the relationship between human beings and technology?* for second-order reflection on values and perceptions concerning the field, aligning with Grin and van der Graaf’s second-order reflection [28].
3. *How to approach synthetic biology ethically?*^1^, in line with Grin and van der Graaf’s *solution-finding* aspect of first-order reflection [28].

An evaluation of these video-narratives [see 30] illustrated their usefulness for reflection on synthetic biology.

This series of clips formed the basis for the three *reflection rounds* of the FRL workshop: the (1) problematization with the first video-clips, (2) further analysis into arguments beyond problematization with the second set of video-clips, and (3) solution finding with the third set of clips. After a general introduction about synthetic biology and frame reflection, the plenary facilitator guided participants throughout the workshop rounds, each comprising a round-specific instruction, the viewing of one set of video-clips, and canvas co-creation exercises. Synthetic biology was introduced by means of a video about the field, based on a presentation used in Betten et al. [31], which aimed to present synthetic biology in a neutral way: as an emerging field that poses known and unknown possibilities and challenges.

**Fig. 2:**
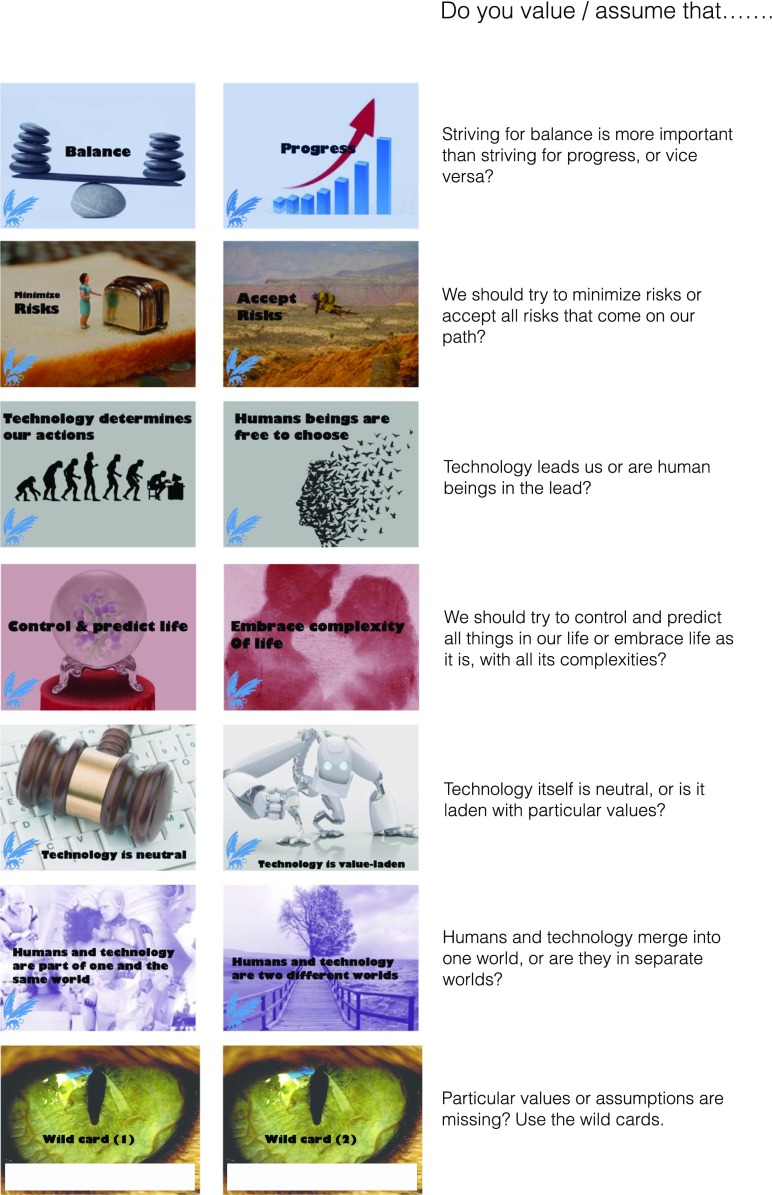
Value & Assumption cards and their intentionally ambiguous meaning

**Fig. 3:**
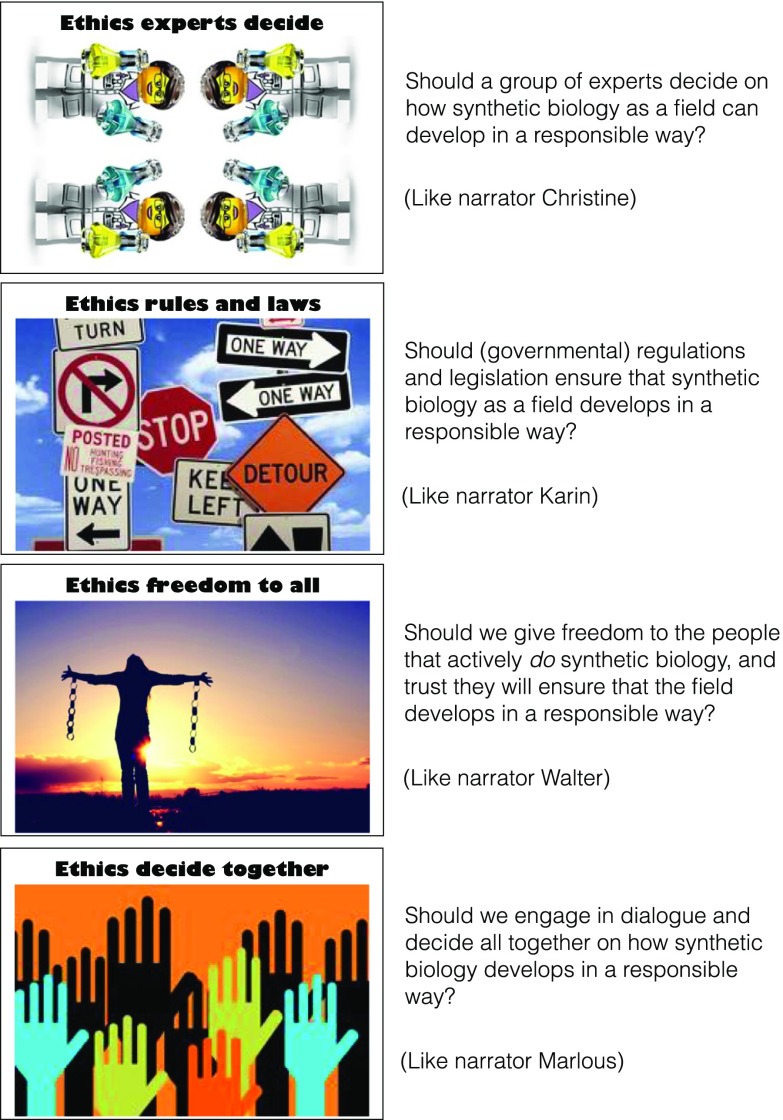
Ethics cards and their meaning

The canvas co-creation exercises corresponded to each workshop round and comprised:

1. Analysis of the first video-clips by means of an A2 canvas with photos of the narrators (see Fig. 1) and markers to write down keywords and draw arrows, which elicit the characteristics of narrators and/or potential differences and similarities between their viewpoints.
2. Identification of more fundamental differences by means of viewing the second set of video-clips and cards about *values* and *assumptions* (hereafter Value & Assumption cards). These Value & Assumption cards were provided in six pairs, recognizable by their similar color, and each contained keywords and an imagination-triggering picture or visualization (Fig. 2): (1) *balance* and *progress*, (2) *minimize risks* and *accept risks*, (3) *technology determines human actions* and *human beings are free to choose*, (4) *control and predict life* and *embrace its complexity*, (5) *technology is neutral* and *technology is value-laden*, and (6) *human beings and technology are part of one world* and *human beings and technology are two different worlds*. Keywords on the cards are value- or assumption-related tension fields that the authors associated with the characteristics of the narrators’ framings as sketched above. Nevertheless, the keywords on the cards were intentionally ambiguous, making it open for FRL participants to decide how the cards and narrators were related in their sense making of the keywords and narratives. We provided two *wild cards* as well, on which participants could write values or assumptions that they considered to be missing in the provided set.
3. Negotiation on an approach to the further development of synthetic biology as a field (step 3), supported by the third set of video-clips and four *ethics cards* (Fig. 3): (1) *experts decide*, (2) *regulations and legislation*, (3) *freedom for all*, and (4) *decide together*. These cards aligned with the views of the narrators as sketched above as follows: (1) Christine, (2) Karin, (3) Walter, and (4) Marlous (see also Fig. 1).

**Fig. 4:**
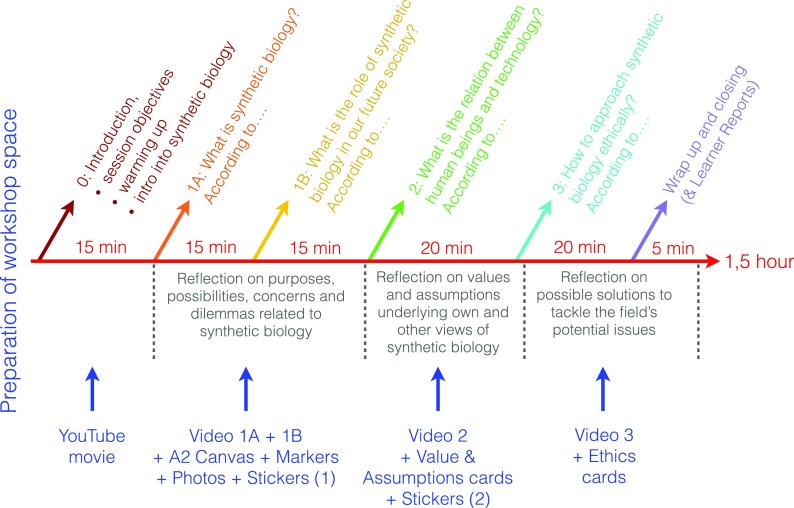
FRL workshop timeline, showing the three workshop rounds, objectives, and used tools per round

**Fig. 5:**
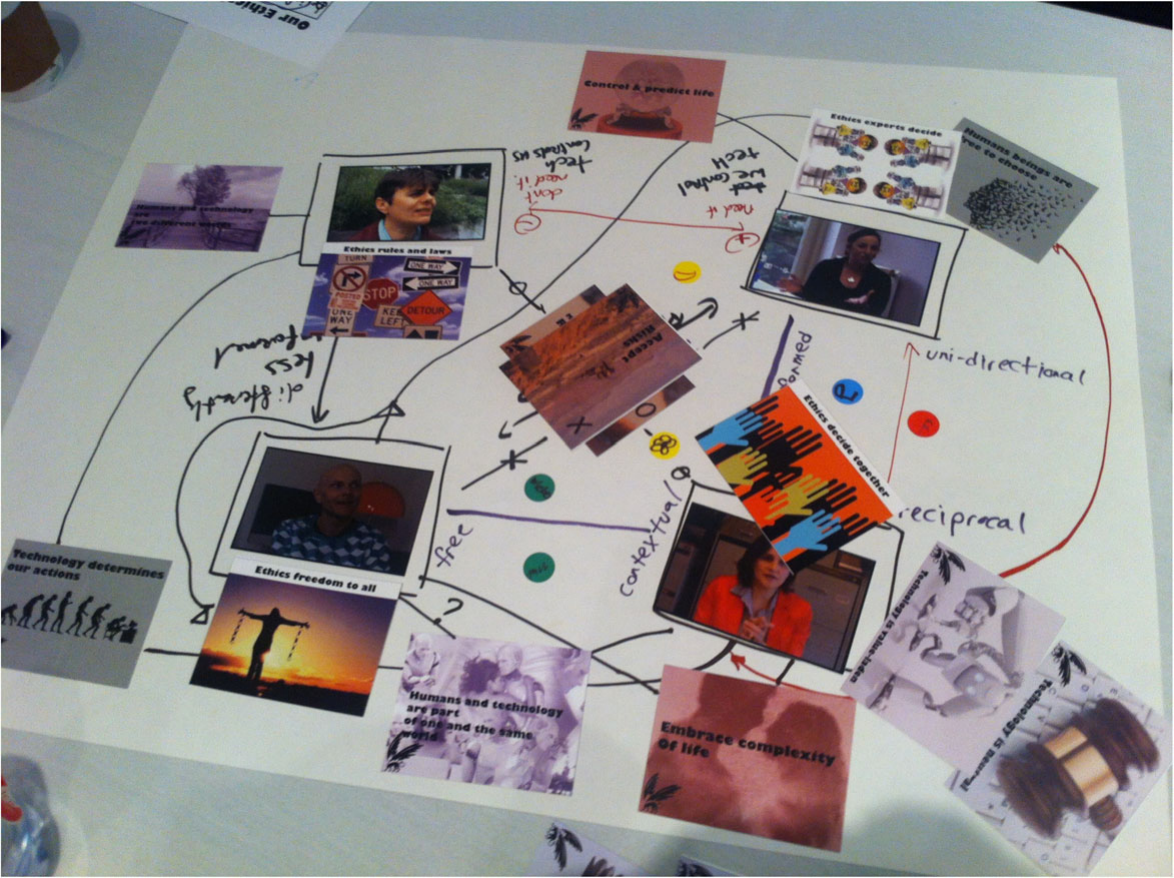
A photo of a canvas created during one of the FRL workshops, to illustrate the co-creation exercises

At the end of steps 1 and 2, participants could indicate their own position in relation to the narrators by placing a sticker on the canvas. In the first round, they annotated a *1* and the first letter of their name on the sticker; in the second round a *2* and the same initial. In step 3, participants were asked to negotiate on the ethical approach to synthetic biology based on the position of their second stickers on the canvas. Although their positions could vary significantly, they had to agree upon a single or combination of ethical approaches to synthetic biology. Figure 4 depicts the workshop timeline that summarizes the different rounds of the FRL. Figure 5 shows the kind of canvasses that were created during FRL workshops.

In each workshop, three additional facilitators walked around to encourage participants to explore differences and similarities in viewpoints, and values and assumptions underlying these, in line with the playfulness process conditions *stimulating guidance*, *experimentation space*, and *focus* (cf. [11]). Participants were allowed to *cheat*: if they did not agree or know what to do during the exercises, they could invent their own ways of doing the exercise.

### Testing the Tool—Data Collection

To collect data on how the FRL supported frame reflection, we organized workshops with a variety of students: three workshops with fifth- and sixth-year high-school students with a biology specialization (*n* = 69), two workshops in Amsterdam with Master students (*n* = 30), and two with synthetic biology Bachelor, Master, and PhD student researchers participating in the international iGem competition during the Giant Jamboree in Boston (*n* = 59). During these workshops, participants were divided into tables of four to seven. Procedural and practical ethical principles of qualitative research were applied (cf. [33]): participation was voluntary, the aims of the study were explained (FRL method evaluation), as well as the participants’ anonymity, and recording began only after having received informed consent. We also informed participants about the fictitious nature of the video-narratives and emphasized that the facilitators were social scientists with no intention of promoting particular viewpoints regarding synthetic biology. Likewise, we asked participants to co-create an environment in which everyone would feel safe to contribute and say what they wanted to.

With the agreement of the workshop participants, we audio-recorded four table conversations of the iGem students, four of the Master students, and four of the high-school students, giving conversation data of 12 tables in total. Also, all workshop participants (*n* = 158) filled out a *Learner Report*. This is a mini-exit survey with open questions intended to monitor insights gained and reflection on one’s own and other people’s viewpoints [34]. The FRL’s Learner Reports contained the following sentences for participants to complete:

- *During this workshop I discovered that my own view on synthetic biology is…*
- *The workshop challenged my assumption that…*
- *The workshop showed me that I particularly value…*
- *During the workshop I saw that other people value/assume…*
- *My view changed/did not change into…*
- *During the workshop I realized that compared to others my view is…*

### Data Processing and Coding

Audio-recorded table conversations were transcribed verbatim. The Learner Report responses and the table conversations were analyzed by a combination of deductive and inductive coding [35] in MAXQDA, on the level of “paragraphs that are connected to a specific context or setting” ([36], p. 137). First, the second author of this study coded the conversations and Learner Report responses with *first-order reflection* and *second-order reflection* as sensitizing concepts [37]:

- *First-order, other*: conversations in which participants discussed a narrators’ evaluation of synthetic biology or the ethical approach to the field in terms of its effectiveness, effects, problems and solutions
- *First-order, self*: participants’ own evaluations with regard to these aspects
- *Second-order, other*: conversations about (estimations of) narrators’ more abstract conceptions such as fundamental preferences (values) or assumptions regarding the social system
- *Second-order, self*: participants’ elicitation of their own fundamental preferences or assumptions

After discussing the first coding round with the first author, in which disagreements were resolved by negotiation between the first and second author, the second author analyzed the conversations again to seek patterns. The first and second author discussed and agreed upon these recognized patterns, and their characteristics were discussed with the fourth author. The first and second author then reconsidered, discussed and revised the new first- and second-order reflection coding in MAXQDA once again. This resulted in the induction of first- and second-order reflection patterns, and several limitations and strengths of the FRL as a method for frame reflection on synthetic biology. Lastly, the first and second authors analyzed the participants’ Learner Report responses to juxtapose the patterns, and in particular participants’ identification of their own first- and second-order reflection. In these Learner Report responses, we identified patterns in: (1) the insights gained into the level of participants’ own and other viewpoints with regard to synthetic biology, (2) discovery of values and assumptions, and (3) views on an ethical approach to synthetic biology.

## Findings

Table 1 provides an overview and explanation of the reflection patterns induced from the analyses. We proceed to detail each pattern in the sections below.

**Table 1:** An overview and explanation of reflection pattern categories and reflection patterns induced from the data analyses

Reflection pattern category	Often occurring patterns	Explanation
First-order reflection		Conversations about the narrators’ or participants’ framings of synthetic biology concerning the field’s effectiveness, (side-)effects and possible solution strategies to deal with these. For example: categorizing framings in terms of being neutral, contra, or pro-synthetic biology in relation to the profession or (estimated) knowledgeability/nescience of the narrator(s).
Second-order reflection		Conversations about the narrators’ or participants’ own framings of synthetic biology in which underlying values or assumptions are considered in relation to the field’s effectiveness, (side-)effects, and solution strategies to deal with these.
	Fill in the blank-style	The keyword of a Value and Assumptions card (or another value or assumption) is mentioned during the conversation but further explicit defining of its meaning remains absent.
	Indirect defining of underlying values or assumptions	The keyword of a Value and Assumptions card (or another value or assumption) is mentioned during the conversation and participants define its meaning by referring to (fragments of) the video-narratives.
	Explicit defining of underlying values or assumptions	The keyword of a Value and Assumptions card (or another value or assumption) is mentioned during the conversation and participants define its meaning through extensive negotiation, whether or not with references to video-narratives.
	Awareness of the process of framing	Participants relate the origin of synthetic biology framing(s) to previous experiences (education, media, etc.).
(Re)framing		Participants develop own viewpoints of or stances towards synthetic biology’s effectiveness, (side-)effects, and solution strategies to deal with these; individually or as a group. Viewpoints evolve over time (during the FRL workshop).
	Diversity	Participants (feel competent to) take unique positions on the A2 canvas, with or amidst photos of narrators. No particular narrator/framing is more popular than others.
	(Minor) viewpoint change	Participants gain insights into other viewpoints, and occasionally incorporate elements of other viewpoints in their own viewpoint. Own viewpoints of participants shift during the session (sticker 1 is on a different spot than sticker 2), occasionally explained with an explicit link to values or assumptions.
	Inclusive convergence on ethical approach to synthetic biology	Participants agree upon a combination of two or three *ethics cards* during their conversation on how to guide the further development of synthetic biology as a field. Participants are open to individual variances regarding this convergence. Well-informed decision-making on the field’s development is emphasized.

### Patterns in First-Order Reflection

In conversations during the first FRL workshop round, after viewing the video-clips about *What is synthetic biology?* and *What is the impact of synthetic biology on society?*, and to lesser extent later on during the workshops, we found 459 instances of first-order reflection. We identified three types of first-order beliefs by which participants analyzed the narrators: (1) the neutrality versus positive or negative stances or tones of the narrators; (2) knowingness and subtlety versus nescience and naivety of narrators, often put in relation to their viewpoints for or against synthetic biology; (3) the narrators’ professions in relation to their synthetic biology viewpoints. The following fragment of a workshop with Master students illustrates the eliciting of first-order beliefs concerning knowledge, in relation to being neutral, pro or contra synthetic biology:

P1: *Is she [Marlous] more neutral?*

P2: *Yes*

P1: *And [Karin] is just, uh…*

P3: *Different*

P1: *Negative*

P3: *She is afraid*

P1: *Cautious*

P3: *And about that scientific article* … *about [plant] resistance [against infections]*… *I think he [Walter] does not even know what resistance means* …

P2: *I think we can also create a knowledge line here?*

P3: *Yes, that too*

P2: *And with Christine?*

P4: *She has a little bit more knowledge* …

The following extract from an iGem workshop illustrates that in their reflections, participants also relate first-order beliefs, e.g., being neutral, for or against synthetic biology, to the narrators’ professions:

P1: *What were their views? She, well the physicist, she is*…

P2: *She seemed very positive*

P3: *She is obviously an engineer*

P4: *All the different narrators related biology to what they do, their professional life or their personal way of thinking*

(*Hummings of yes*)

P1: *Yeah coz like, it seems, she’s a scientist, she does it all the time. She’s a teacher, and she thinks that you know* ….

P3: *I don’t think that she’s a teacher*

P4: *No, she’s a journalist*

From both extracts it can be deduced that participants categorized the narrators based on beliefs that were grounded in their ideas about the narrators’ characteristics: in the first extract they related being extremely critical or extremely enthusiastic about synthetic biology to having little knowledge, and being neutral to or conscious about the field’s potential opportunities to being knowledgeable; in the second abstract, participants assumed that a physicist (narrator Christine) is more knowledgeable than, for instance, a teacher (narrator Karin), and therefore conscious about the opportunities of synthetic biology. In the instances of first-order reflection, participants merely used words that narrators had themselves mentioned in the video-clips. Formulation and analysis in their own words was often lacking. This first-order reflection continued after the first workshop rounds. For example, in conversations about the ethical approach to synthetic biology (workshop round three), participants occasionally re-emphasized the role of knowledge in relation to stances towards synthetic biology, or even being eligible to have a say in the field, albeit far less frequently than in the earlier workshops.

### Patterns in Second-Order Reflection

Second-order discourse was often, but not entirely, triggered after the second workshop round in which participants watched video-clips about *What is the relationship between human beings and technology?* and did the Value & Assumption card-sorting exercise. The second-order reflection became more profound in the second and third round of the workshop. We identified four patterns in the 180 instances of second-order reflection with various levels of depth, which we describe and illustrate below.

The basic level of second-order reflection could be characterized as a *fill in the blank*-style, in which participants briefly attributed the keywords described on Value & Assumption cards, placing them on the table between or close to particular narrators, but with little argument or explanation. The following extract from a conversation about “which card should be where on the canvas” (e.g., Fig. 5) between high-school students illustrates this:

P1: *Accept risks*

P2: *That’s what this one [Christine] says.*

P3: *No he [Walter] accepts the risks*… *Yes both.*

P4: *She [Christine] doesn’t, she’s just*…

P3: *Yes, she [Christine].*

P1: *Yes, she [Christine] also said that rather a lot.*

P3: *Yes, she [Christine] accepts them partially.*

P1: *Ok, neat [card is placed between them both].*

As this conversation about the Value & Assumption card *accepting risks* shows, this type of negotiation touches second-order reflection. It covers reflection on a value or an assumption (e.g., as suggested by a participant, or written on a card), in this case accepting that technological innovation comes along with risks, but not very profoundly. Of the 180 instances of second-order reflection, this pattern occurred 67 times, of which half occurred in the first workshop round, before the card-sorting exercise.

Another style of second-order reflection concerned the indirect defining of a value or an assumption (mostly as written on a card) by means of reasoning whether and how this particular value or assumption would fit to one or more particular characters. In this, participants often referred to narrators’ words from the video-narratives. The following extract of a workshop with Master students illustrates this:

P1: *Technology Determines our [actions], perhaps a bit with Karin.*

P2: *That is more a keyword for Marlous. Yes Yes. For she says*… *In parallel worlds. I think it suits every character.*

P1: *Marlous said that synthetic biology influenced our society and vice versa*…*.*

P3: *This is also something neutral. It’s not positive or negative.*

P1: *She [Karin] is also very much like: everyone is “connected”; calling it the “head down generation.”*

Facilitator: *In a certain way it fits with Karin and Marlous?*

All: *Yes.*

The participants defined the Value & Assumption card *Technology determines our actions* by referring to the words of the narrators in the video-narratives. In the 75 instances that this pattern occurred, of which 75% were in the second and third workshop rounds, participants often went *deeper* by providing more of own interpretations of the video-narratives in addition to the references to literal words or sentences of the narrators.

A more extensive form of second-order reflection took place when participants explicitly defined the Value & Assumption cards, explored various interpretations, and created explicit references to the narrators’ words, whether or not accompanied with their own interpretations of these, and thereby negotiated the position of the cards on the canvas. The following fragment of an iGem workshop illustrates this:

P1: *And balance?*

P2: *Where is the balance card?*

P3: *You have it.*

P2: *Yes, but you see, what is balance? What is your interpretation of it? He [Walter] says about balance: things will invent themselves.*

P4: *It’s more about balance like with the technology we try to decide together on the speed by which it should develop.*

P5: *I don’t think this [pointing to the card of Walter] is balance, really; he says it [humans and technology] is all the same, and if that’s the same, we cannot really have balance.*

P2: *And what she [Christine] says, like humans do not always have control over what they invent but do have control over what they do with it. That’s a kind of balance too.*

P6: *We have to put them somewhere, otherwise we won’t have enough time.*

P2: *Ok so here [between both narrators].* (*Hummings of agreement*)

As this extract illustrates, the participants co-created and negotiated on the meaning of balance, after which they conclusively agreed upon where to place the card on the map. This pattern, which occurred 20 times almost predominantly in the second and third workshop rounds, often made participants compare one or multiple narrators in relation to their own viewpoint(s), to make overarching or deeper commonalities and differences more explicit.

A last pattern that we identified (18 instances) concerned a process in which participants elicited awareness of their own ways of learning, either during or beyond the workshop (studies, general life). The following extract from a Master student workshop during the third workshop round, when participants decide which ethical approach they would like to take to synthetic biology, illustrates this:

Facilitator: *So, you have agreed upon ethics “Decide together?”*

P1: *Because*…*. We are studying this!*

P2: *We choose “Experts decide” and “Decide together.”*

P1: *We are indoctrinated!*

Especially in Master students’ workshops, participants expressed awareness of their background in their reflection (14 of the 18 instances). In their Master specialization Management, Policy & Entrepreneurship, and Global Health, major attention is paid to epistemic cultures (positivism, constructivism), and the value of multi-stakeholder processes. This may have raised their awareness of the impact of their own studies on their stance to the preferred development of emerging fields like synthetic biology. iGem workshops showed only two instances of this reflection pattern, as did the high-school workshops.

### Patterns in Participants’ (Re)framing of Synthetic Biology

Analysis of conversations during sticker moments, which took place before and after the reflection on values and assumptions (see prototype description), showed that a great diversity of viewpoints was represented in most workshops. In 13 instances participants said feeling too ignorant to form an opinion during the workshops, but, generally speaking, most participants did position themselves close to one particular narrator or between two or more narrators. No particular narrator was more popular than another. Nor did the table participants place their stickers all together in one spot. Comparing the two sticker moments, many participants placed their second sticker in a slightly different spot on the canvas than their first one, indicating that they had made minor changes. This replacement was occasionally accompanied by the eliciting of arguments for their change in position during the table conversations, in which several instances of second-order reflection could be identified, for example, a reference to values and assumptions that had caused their change of position. We illustrate this with the following extract from a high-school workshop participant in conversation with a facilitator, during the sticker moment after reflection on the relationship between human beings and technology:

P1: *Ah, I had a hard time to understand her [Christine] before, but now I understand her better. Can I have a marker?*

Facilitator: *You said you did not understand her earlier?*…

P1: *She [Marlous] is a little bit too accepting. I want a middle ground. And she [Christine] does that.*

Facilitator: *She [Marlous] is too accepting?*

P1: *(…) She says [Christine] “one should accept risks, but also control it [synthetic biology] well.” At least, that is what I got out of it. She [Marlous] is more like “we should do it all.”*

The participant discovers a change in the own preferred position on the canvas (in relation to the narrators), by reconsidering the degree of control on synthetic biology as a field. Apparently, the participant argues that there is a more open stance on Marlous’ side of the canvas, where this participant initially positioned the own viewpoint, versus a more controlling stance on Christine’s of the canvas. As this participant suddenly realizes the own preference for more control, the participant “moves” to a new location on the canvas, closer to Christine.

Although explicit reference to participants’ own values and assumptions was not so common during conversations about their own viewpoints regarding synthetic biology, the participants’ responses to the Learner Report questions showed something different. For instance, in these answers, many participants emphasized that they had gained insights into their own and other people’s values and assumptions related to their own and other views of synthetic biology during the workshop. These additional data show that the participants themselves recognized second-order reflection too, albeit in their own words. Participants’ responses given in the Learner Reports to the fill-out sentence “My view did/did not change into…” confirm our idea that most participants slightly changed their viewpoints. Of the 126 participants who filled out this question, about half explicitly reported not or “not really” having changed their view. Sixteen noted having *changed* from *no view* to *a view*, and another 20 explicitly reported gaining new insights and making minor changes, for example as an iGem participant reported having changed his view “into a more flexible one, with wider perspective on the issues.” In other words, many participants seemed conscious that they had become more aware of other viewpoints, their embracing of differences arose, or (occasionally) that they had incorporated different elements of other viewpoints in their own framing.

### Patterns in (Re)framing the Ethical Approach to Synthetic Biology

During the negotiation about the ethical approach to synthetic biology at the end of the workshop, most tables reached a consensus on a combination of letting *Experts decide* (going along with narrator Christine) on the ethical approach to synthetic biology and *Decide together* (narrator Marlous). This negotiation often concerned a discussion about *what is an expert?* The following abstract of a high-school workshop illustrates this:

P1: *What would you choose?*

P2: *What would I choose? I think a sort of committee [of experts] but the citizens should have a little bit of a say too.*

P3: *But wait, she was talking about scientists! With their “Erlenmeyer’s” in the lab.*

P2: *I think there must be some kind of mix. It’s logical that a sort of knowledgeable group of people decides what is allowed and not, and that citizens have a say too. (…) Because (*…*) my parents might know better what is good for me and what not, but that does not mean that I (*…*) agree with everything they say. I want to have a say too (*…*)*

Facilitator: *Let’s see* … *you [participant P4] are in that corner [with Walter], I guess? And do you think that [“Freedom to all”] too? (*…*)*

P4: *Yes. But I think through this [exercise] I moved a little bit more to these two [“Decide together” and let “Experts decide”]. Because, it [synthetic biology] must be reviewed by people who really know something about it, but I’m still progressive over all, because the development [of synthetic biology] must happen and is a logical step.*

The groups chose the ethical approach with *Rules and laws* (narrator Karin) less frequently and mostly in combination with *Experts decide* or *Decide together* and often accompanied with a discussion about *who decides these rules? Freedom to all* as an ethical approach (narrator Walter) was unpopular in all workshops. In the occasional instances that one group member’s sticker was positioned *far away* from all the others on the canvas, e.g., at the location of the *Freedom to all-*card with Walter, he or she would often agree with the group’s choice after the negotiation. In other words, the group mostly came to a unified stance towards the ethical approach for responsible development of synthetic biology, in which openness seemed to remain for different stances towards the field itself.

In the Learner Report responses, many participants addressed their view of an ethical approach to synthetic biology as an important insight that they had discovered during the workshop (69 instances). Relatively speaking, many of the iGem participants (40 instances of 78 who filled out the Learner Report) emphasized the importance of knowledge in deciding how synthetic biology can develop and the need for reason in deciding on the future of synthetic biology. Of the 69 participants who addressed the ethical approach in their Learner Report, 25 called for experts to decide on the ethical approach to synthetic biology, whereas 27 participants re-emphasized the need to allow many stakeholders or citizens decide upon it together. So, although many participants argued that well-informed decision-making on the future of synthetic biology is important, there was no consensus that the well-informed people taking such decisions should be the experts in the synthetic biology field itself.

## Discussion

Our findings illustrate that a playful method like the FRL is suited to facilitating frame reflection on an emerging and controversial field such as synthetic biology with different social groups.

Although the conversations in FRL test workshops at high schools were somewhat less thorough than the workshops with iGem and Master students, we identified a great deal of second-order reflection throughout all workshops. This included instances of the more profound versions of second-order reflection, in which participants explicitly defined a relevant value or assumption (e.g., as written on Value & Assumption cards), extensively discussed the narrator of the videos who adhered to this value or assumption and why, and/or elaborated on their own stance in relation to that. Although the high-school participants were enrolled in the highest level of Dutch secondary education (Gymnasium), we hypothesize that playful process conditions and activities as embedded in the FRL method could readily support frame reflection among different social groups. Further research into playful reflection methods comparable to the FRL, with ever more diverse participants, is needed to establish whether they support frame reflection among adults with no academic education and high-school students from other educational levels. If there is a need for comparable methods for playful RRI reflection, about synthetic biology or other fields, several of our findings may be worthy of consideration.

First, a study by van der Meij et al. [30] into reflection on synthetic biology that used the same video-narratives as used in the FRL, but no other tools, concluded that video-narratives are not sufficient to achieve reflection on values and assumptions. The authors concluded that narrative-based reflection needs to be complemented with strong facilitation and conversation-triggering cards to deepen the reflection to a second-order level (ibid.). Indeed, in the FRL, we saw that the use of the Value & Assumption cards placed greater emphasis on second-order reflection than could be seen in van der Meij et al. [30]. The keywords on the Value & Assumption cards, accompanied by ambiguous visualizations, allowed for certain freedom of interpretation (see Fig. 2). The developers of the FRL, first and last authors of this study, had formulated the keywords based on value or assumption-related tension fields that they associated with the characteristics of the narrators’ views (cf. [30]). Furthermore, the card designs were based on playfulness principle *imagination* [11]. In accordance with this playfulness design principle, we noted that the cards helped to focus the conversations among the FRL participants, while their freedom of interpretation seemed to stimulate the more profound forms of second-order reflection. Authors of a study into a card-based nanotechnology reflection method [7] also noted that if participants could use keyword cards in a rather open manner, this contributed to the development of ownership during the reflection (finding one’s own words as opposed to using expert vocabulary). We therefore argue that (1) the use of cards with second-order keywords and (2) images with certain interpretative freedom, in addition to (3) narratives (in any form) and (4) analysis exercises, is a combination worth considering in designing reflection methods, especially if the cards and narratives are carefully chosen in line with a framework for dominant frames of the relevant emerging technology.

Second, van der Meij et al. [30] also found that video-narratives can trigger annoyance with a narrator, which calls for careful consideration of video-narrative design and presentation. Adding to the findings of van der Meij et al. [30], we noted that our FRL participants expressed first-order judgmental assumptions regarding the narrators’ knowledge about and view of synthetic biology, e.g., due to their (fictitious) profession. Horst and Michael [15] could have called these immediate judgments of our FRL participants *idiotic behaviors* (p. 283), which often say much about what we overlook in designing tools for reflection on science or technology. It is apparent and indeed logical that people have many preconceptions about what *kind of people* have the *right knowledge*, *whom to trust* and *what kind of people have which opinions*. In our search for good narratives to stimulate reflection on synthetic biology, we overlooked the consequences of contextual elements in the narratives, like details about the (fictitious) professions of the narrators, for the reflection. Our study calls for more research into the balance between providing contextually rich video-narratives, which are identifiable and realistic for participants of RRI reflection processes, and the triggering of (and dealing with) superficial judgment in the analysis of viewpoints represented in the narratives. Van der Meij et al. [30] noted that the narrators should be presented as equally knowledgeable. Based on this study we could add that creating a certain *mystery* about the narrators’ personal lives could also be an interesting direction.

Third, if second-order reflection on research and innovation is desired in RRI reflection, our study showed that it is not enough to use playful activity principles in the design of playful tools. Our findings indicate that particular forms of facilitation are necessary, particularly to encourage second-order reflection. We noted that about one third of the instances of second-order reflection during FRL workshops occurred when a facilitator joined the table conversations. The facilitators who walked around during FRL workshops visited the various tables and asked participants to recap their conversations. Facilitators mostly asked *why?* questions in response to the recapitulation. This style of facilitation in particular contributed to second-order reflection. Given the potential expense of large-scale workshops with many tables, we suggest that facilitation tasks might better be divided among participants themselves. The plenary facilitator could instruct the chair of each table to ensure the reflection structure.

There might also be a need for comforting facilitation to make playfulness design elements *imagination* and *experimentation space* more productive in achieving second-order reflection in playful RRI reflection methods. The FRL provided participants with tools characterized by a certain freedom of interpretation, especially in the Value & Assumption cards with keywords and suggestive pictures, and by permitting participants to *cheat* during the exercises. As described above, the ambiguity of the tools obliged participants to negotiate about the meaning of the keywords and pictures, and after which, they often started to analyze narrator viewpoints extensively, e.g., on a second order, to define the position of the card in the canvas. On the other hand, the ambiguity of the Value & Assumption cards could inhibit reflection as well. For example, one participant of a high-school workshop said: “Huh? A huge piece of bread, a really small woman, and a huge toaster…? How can this be ‘minimize risk’?,” after which the discussion on this card was rather blocked, and the participants had to ask the facilitator whether they were *doing it right*. Considering such instances of insecurity, one conclusion might be that the tools of the FRL method were too complex for e.g. high-school students. However, in this and various other instances of insecurity about the cards and their keywords during the workshops, a facilitator’s re-emphasis that all interpretations were fine often restored participants’ confidence. Therefore, we argue that ambiguity is productive for frame reflection, especially for second-order reflection, if additional comforting facilitation is offered during the participants’ negotiation of meaning surrounding the ambiguity.

Fourth, a potential point of investigation is the further use of co-creation exercises in RRI-related reflection methods. Felt et al. discourage the use of a consensus exercise, for it may “lead to a premature reduction of the scope of opinions” ([7], p. 236). Our last exercise in the FRL, however, asked participants to reach a consensus on the ethical approach to synthetic biology. We based this exercise on the notion that co-creation is a good way to make people negotiate and thereby discover a diverse set of viewpoints [11]. As we identified that the reflection during this consensus building actually allowed space for diversity, further research into comparable exercises is needed to assess whether asking people to reach value- or assumption-based consensus (on the ethical approach to the field or in other areas) impedes or assists the sharing of diverse viewpoints.

## Conclusion

This study shows that the Frame Reflection Lab (FRL) method encouraged frame reflection in various ways. Analysis of the workshop conversations showed that in addition to first-order reflection, there were many instances of second-order reflection. Participants analyzed the views represented in the video-clips at a deeper level than simply as problems and solutions surrounding synthetic biology. They really delved into the narratives of the videos and came to a deeper understanding of the values and assumptions underlying the narrators’ views. At the beginning of the workshops, participants mostly expressed more first-order beliefs, such as judgments surrounding the narrators’ neutrality/bias, knowledge or professional status (scientist, arts teacher, music producer, journalist). Later on, their reflections on video-clips about the relationship between human beings and technology, supported by the Values & Assumptions card-sorting exercise, triggered explicit conversations about values and assumptions, leading to deeper analysis of differences and commonalities between viewpoints. Although many participants reported in their Learner Reports having stayed close to their initial view of synthetic biology, we observed that their openness to alternatives changed during the workshops. For example, we saw greater awareness and occasional appreciation of other people’s stances towards synthetic biology based on the analysis of viewpoints and their underlying values and assumptions.

### Towards a Kaleidoscope of Reflection Methods

Davies et al. [14] pointed out that each public engagement format has its own strengths and weaknesses in terms of reaching openness for diversity, emotional engagement, and scientific accuracy. As it might be better to see “public participation as comprised of a kaleidoscope of practices rather than a single static event” ([14], p. 356), we argue that the FRL should not be seen as a free-standing exercise in the RRI spectrum, but should ideally be embedded in a wider context of RRI reflection. For example, in RRI it can be useful to reflect on specific applications, which the FRL does not currently facilitate. Kerbe and Schmidt found that citizens tend to make a ranking in hierarchies of organisms, such as bacteria, pigeons, horses and human beings [16]. They allow more degrees of “synthetic-ness” among bacteria than in higher organisms (ibid.). They also noted that visitors preferred certain boundaries for the latter, while the former was allowed more freedom (ibid.). With this in mind, it could be interesting to do an FRL workshop before more case-specific reflection on particular synthetic biology applications.

The FRL could also possibly be an interesting strategy to tackle deadlocked policy discussions in which the involved actors fail to grasp what causes their impasse. In such cases, engaging in a frame reflective conversation by means of the FRL could help in identifying values and assumptions that underlie the rationales for the different viewpoints. In that way, we argue, actors may find common values that could even lead to resolving the deadlock.

Furthermore, the FRL method could function as a tool for collecting systematic qualitative data on viewpoints regarding synthetic biology, comparable to Felt et al.’s card-based method for the social dialogue on nanotechnology [7]. Our intention in designing the FRL was more to investigate it as a playful method for reflection, but we have seen that the method has the potential to structurally collect data, e.g., about people’s views of synthetic biology for policy-making, that goes beyond the method as described in Felt et al. [7]. For instance, in addition to collecting views on being enthusiastic about or hesitant towards synthetic biology, the FRL method also reveals people’s reasons behind their viewpoints. These insights can enrich the knowledge about concerns, hopes, and dreams of citizens or stakeholders that should be the entry point for inclusive deliberation and action in the further development of (policies for) the field of synthetic biology.

## Footnotes

1 The words *ethical* and *moral* are often used interchangeably. This question refers to what could be called a *moral approach*: *What is the adequate approach to deal with the development of synthetic biology as a field?* The makers of the video-clips decided to use the more compact formulation *How to approach synthetic biology ethically?* for readability purposes.
